# Spinal cord anomalies in children with anorectal malformations: a retrospective cohort study

**DOI:** 10.1007/s00383-023-05440-y

**Published:** 2023-03-19

**Authors:** Cunera M. C. de Beaufort, Julia C. Groenveld, Tara M. Mackay, K. Mariam Slot, Sjoerd A. de Beer, Justin R. de Jong, Joost van Schuppen, Carola J. McDonald, Dewi P. Bakker, Elske van den Berg, Caroline F. Kuijper, Ramon R. Gorter

**Affiliations:** 1 Department of Pediatric Surgery, Emma Children’s Hospital Amsterdam UMC, Location University of Amsterdam, Meibergdreef 9, Amsterdam, The Netherlands; 2 Amsterdam UMC, Gastroenterology and Hepatology, Amsterdam Gastroenterology Endocrinology Metabolism, University of Amsterdam, Meibergdreef 9, Amsterdam, The Netherlands; 3 Amsterdam UMC, University of Amsterdam, Amsterdam Reproduction and Development Research Institute, Amsterdam, The Netherlands; 4 Amsterdam UMC, Department of Surgery, Location University of Amsterdam, Meibergdreef 9, Amsterdam, the Netherlands; 5 Amsterdam UMC, Department of Neurosurgery, Location University of Amsterdam, Meibergdreef 9, Amsterdam, the Netherlands; 6 Amsterdam UMC, Department of Radiology and Nuclear Medicine, Location University of Amsterdam, Meibergdreef 9, Amsterdam, the Netherlands; 7 Amsterdam UMC, Department of Pediatric Neurology, Location University of Amsterdam, Meibergdreef 9, Amsterdam, the Netherlands; 8 Department of Pediatric Urology, Emma Children’s Hospital Amsterdam UMC, Location University of Amsterdam, Meibergdreef 9, Amsterdam, the Netherlands; 9 Department of Pediatric Surgery, Emma Children’s Hospital Amsterdam UMC, Location University of Amsterdam, Amsterdam Gastroenterology and Metabolism Research Institute and Amsterdam Reproduction and Development Research Institute, Meibergdreef 9, 1105 AZ Amsterdam, The Netherlands

**Keywords:** Anorectal malformations, Spinal cord anomalies, Tethered spinal cord, Untethering surgery, Children

## Abstract

**Purpose:**

First, to assess the number of spinal cord anomalies (SCA), specifically tethered spinal cord (TSC) in patients with anorectal malformations (ARM), identified with spinal cord imaging (i.e. spinal cord US and/or MRI). Second, to report outcomes after TSC treatment.

**Methods:**

A retrospective mono-center study was performed. All ARM patients born between January 2000 and December 2021 were included. Screening for SCA consisted of spinal cord US and/or MRI. Radiology reports were scored on presence of SCA. Data were presented with descriptive statistics.

**Results:**

In total, 254 patients were eligible for inclusion, of whom 234 (92.1%) underwent spinal cord imaging. In total, 52 (22.2%) patients had a SCA, diagnosed with US (n = 20, 38.5%), MRI (n = 10, 19.2%), or both US and MRI (n = 22, 42.3%), of whom 12 (23.5%) with simple, 27 (52.7%) intermediate, and 12 (23.5%) complex ARM types. TSC was identified in 19 patients (8.1%), of whom 4 (21.1%) underwent uncomplicated neurosurgical intervention.

**Conclusions:**

SCA were present in 22% of ARM patients both in simple, as well as more complex ARM types. TSC was present in 19 patients with SCA, of whom 4 underwent uncomplicated neurosurgical intervention. Therefore, screening for SCA seems to be important for all ARM patients, regardless of ARM type.

**Level of Evidence:**

Level III.

## Introduction

Anorectal malformations (ARM) are rare congenital disorders that occur in approximately 1–3 per 5000 live births yearly [1]. ARM are classified according to Krickenbeck classification, and different types exist, varying between relative simple (e.g., recto-perineal fistula) and more complex types (e.g., recto-vesical fistula or cloacal malformations) [2]. In approximately 60% of ARM patients, additional anomalies occur [3–5]. ARM may be part of the VACTERL-association (Vertebral, Anorectal, Cardiac, Tracheo-Esophageal, Renal, and Limb anomalies) or other genetic syndromes. [6–8]. Another important tract in which additional anomalies in ARM patients might be present is the spinal cord [9, 10]. There is a wide variety and range of severity of spinal cord anomalies (SCA) that can be present in ARM patients, such as tethered spinal cord (TSC), spinal lipoma, or syringomyelia. In patients with ARM, TSC might be associated neurological problems, at young and/or later age, such as neurogenic bladder or development of walking disorders [11, 12]. In patients with TSC, the spinal cord is caudally attached to abnormally inelastic structures (e.g., fatty or fibrous terminal filum (FT), meningocele, spinal lipoma, or tumors) [13, 14]. Patients with TSC can be asymptomatic, but especially during growth when the myelum should move freely within the bony vertebral column, symptoms as pain, stiffness, gait deformities due to muscle weakness, sensibility anomalies of the legs and urinary problems (e.g., neurogenic bladder) can occur [10, 13]. Because SCA are often present in ARM patients, it should be an item of discussion to add SCA as a factor to the VACTERL-association [15]. However, the exact prevalence of SCA in ARM patients remains unclear [3, 14].

Early identification of SCA in ARM patients is important, as their presence could potentially cause damage in the future when not recognized (e.g., neurogenic bladder disorder in patients with TSC that might result in vesico-urethral reflux, impaired kidney function or urinary incontinence) [16, 17]. Therefore, screening for SCA in all ARM patients is recommended [9]. In current practice, discussion remains on the optimal imaging study for identification of SCA [13, 18, 19]. Although magnetic resonance imaging (MRI) of the spinal cord is accepted as golden standard for identification, an ultrasound (US) of the spinal cord might also be a reliable and a quicker alternative imaging modality with lower costs [19]. However, due to bone formation later on in life, the spinal cord is difficult to visualize with US if performed after the neonatal period (i.e. within 3 months after birth) [20].

Although most patients with SCA (including TSC) do not require surgical intervention, there is an ongoing debate on whether or not an early neurosurgical intervention (i.e. prophylactic untethering surgery) is beneficial for ARM patients [21, 22]. A strict indication to perform neurosurgical intervention is presence of neurological symptoms (e.g. pain, motor and/or sensory deficit, bladder and/or bowel dysfunction). However in ARM patients, problems such as neurogenic bladder disorder or fecal incontinence (both due to anatomical impaired sphincter function) are common and might not improve after untethering [16, 23]. Furthermore, as with all surgical interventions, complications might occur. Therefore, the aim of this study is twofold. First, we aimed to assess the number of SCA, specifically TSC in ARM patients, identified with spinal cord imaging (i.e. spinal cord US and/or MRI). Second, we aimed to report the outcome of the treatment strategy for TSC in our children with ARM.

## Methods

### Study design and patient population

This was a retrospective cohort study, designed in accordance with the Strengthening the Reporting of Observational Studies in Epidemiology (STROBE) guidelines [24]. Data were retrieved from a retrospectively maintained database (using Castor EDC (Electronic Data Capture) software [25]) including newborns with any type of ARM (according to Krickenbeck classification [2]), who were born between January 2000 and December 2021 in AmsterdamUMC, or ARM patients referred to AmsterdamUMC at later age. This study was performed at the Emma Children’s hospital, Amsterdam University Medical Center (AmsterdamUMC), a tertiary referral center, accredited by the national authority as one of the centers of expertise for ARM in the Netherlands, and a member of European Reference Network (ERN) eUROGEN. Eligible for inclusion were all patients from the retrospectively maintained database. For this study, patients who deceased within one day after birth were excluded. Follow-up was calculated from date of birth to latest hospital visit.

### Ethics

This study was reviewed by the medical ethical commission and was not subject to the WMO statement (ref. no. W20_230 #20.576). Regarding the primary Emma Children’s hospital database, written information was provided to parents or legal guardians for all identified ARM patients, including a letter of objection. In case of objection, patients were removed from the database (n = 6).

### Data extraction

Data on patient characteristics, (non-)syndromic anomalies (e.g., VACTERL-association, Cat-eye syndrome, and Townes-Brocks syndrome), type of ARM (according to Krickenbeck classification [2]), additional anomalies (i.e. vertebral and SCA) associated symptoms (e.g., neurogenic bladder, impaired bowel function, abnormal reflexes, and gait abnormalities), and additional imaging studies (i.e. US, vertebral x-ray, and MRI) were extracted from the database by 2 independent researchers (JG, CdB). Furthermore, consultation (i.e. from a pediatric urologist, neurologist, and neurosurgeon), presence and type of neurosurgical intervention for SCA (e.g., untethering surgery in case of TSC), and postoperative complications were extracted from the database. In case of discrepancies in data extraction, an expert panel, consisting of a pediatric surgeon (RG), pediatric urologist (CK) and pediatric neurosurgeon (MS), was consulted for final decision.

### Definitions

Patients were classified according to their type of ARM using the Krickenbeck classification with major clinical groups and rare/regional variants [2]. For statistical analysis, ARM types were further classified into 3 categories: simple (i.e. recto-perineal fistula and anal stenosis), intermediate (i.e. recto-vestibular, recto-urethral and no fistula), and complex types (i.e. recto-vesical fistula, cloacal anomalies and rare/regional variants). Radiology reports were scored on presence of SCA. No imaging was repeated. SCA were defined as any congenital defect of the spinal cord including the spectrum of spinal dysraphisms. According to AmsterdamUMC protocol, all children with ARM should undergo a spinal x-ray and US as part of the ‘V’ in VACTERL screening. Subsequently, in ARM patients aged younger than 3 months, US of the lumbar myelum was primarily performed for SCA identification. In case anomalies or uncertainties were found with US, additional MRI was performed. In children aged older than 3 months, MRI was primarily performed for SCA identification due to the ossification of the sacral and lumbar spine around that age [20]. The presence of TSC, was defined as radiological TSC with or without symptoms. Definitive TSC diagnosis was at the discretion of a pediatric neurologist, neurosurgeon and/or pediatric radiologist, and defined as conus medullaris position below L2, in combination with signs of fatty FT or tight FT and reduced movements of the conus on US. Back pain, leg pain, feet deformities, impaired urinary function (e.g., neurogenic bladder dysfunction), and abnormalities through neurological examination (e.g., gait abnormalities, scoliosis, abnormal reflexes and sensitivity, performed by a pediatric neurologist) were considered symptoms associated to TSC. Vertebral anomalies (diagnosed with a spinal x-ray and/or MRI) were classified into segmentation, formation or combined defects. In case of uncertainties regarding classification of additional anomalies and symptoms, an expert panel consisting of a pediatric radiologist, pediatric urologist, pediatric neurologist (within the expertise center for spina bifida), pediatric neurosurgeon, and pediatric surgeon were consulted.

### Outcomes

Primary outcome was the number of ARM patients in whom SCA were identified.

Secondary outcomes were the number of US and/or MRI performed, the screening trend over time, factors associated with SCA, the number of ARM patients with TSC, and the number of ARM patients with symptomatic or asymptomatic TSC that underwent treatment and their functional outcomes.

### Statistical analyses

Statistical analysis was conducted using IBM SPSS Statistics for Windows, Version 28 (IBM Corp., Armonk, N.Y., USA). Descriptive statistics were used for analysis of baseline characteristics. These were reported as proportions and percentages for binary or categorical variables, and as mean with standard deviation (SD) or as median with interquartile range (IQR) for continuous variables as appropriate. To evaluate changes over time in screening, patients were categorized into 3 time periods (i.e. period 1: 2000–2006, period 2: 2007–2014, period 3: 2015–2021). To compare screening percentages over time, Chi-square for trend was used. Univariable and multivariable logistic regression analyses were performed to investigate associations between baseline characteristics (i.e. sexe, presence of syndromes, presence of vertebral anomalies, and type of ARM (simple vs. intermediate vs. complex) and SCA. Outcomes were reported as odds ratio (OR) with corresponding 95% confidence interval (95% CI). A p-value of < 0.05 was considered statistically significant. Missing or unknown data were described. In case of missing data in additional imaging studies (US and MRI), it was classified as ‘not performed’.

## Results

### Participants

In total, 255 patients were identified in the Castor EDC database of whom 1 was excluded due to death within 1 day after birth. The cohort therefore comprised of 254 patients (n = 85 in period 1, n = 84 in period 2, n = 85 in period 3); 122 female (48.0%) and 132 male patients (52.0%), with a median age of 7.0 years (IQR 3.0–12.0) at latest follow-up. Eight different ARM types were identified (simple n = 115, intermediate n = 107, and complex n = 29), of which recto-perineal fistula occurred most often, encompassing 51 female patients (41.8%) and 58 male patients (43.9%). In 82 patients (32.3%), VACTERL-association (n = 42 (16.5%)) or syndromic anomalies (n = 40 (15.7%)) were identified. Nine patients (3.5%) deceased during the study period, of which 3 patients (33.3%) had a cloacal anomaly. Five patients died due to respiratory insufficiency (at ages 2 days, 3 days, 2 months, 3 months and 31 months, respectively), 1 due to bacterial meningitis (at age 3 years), and 1 due to abdominal compartment syndrome (at age 16 years). In 2 patients, the cause of death was not mentioned in the available medical record. A complete overview of identified ARM types is shown in Table 1.

**Table 1:** Baseline characteristics

Type of ARM	Sex		Age (years)	Anomalies	
	Female	Male	At latest follow-up	VACTERL or syndromic*	Vertebral
	n (%)	n (%)	median (IQR)	n (%)	n (%)
Simple					
Recto-perineal fistula, n = 109	51 (41.8)	58 (43.9)	5.0 (2.0–10.0)	24 (29.3)	10 (9.2)
Anal stenosis, n = 6	3 (2.5)	3 (2.3)	7.0 (1.5–12.5)	3 (3.7)	1 (16.7)
Intermediate complex					
Recto-vestibular fistula, n = 49	49 (40.2)	NA	7.0 (5.5–12.0)	15 (18.3)	11 (22.4)
Recto-urethral fistula, n = 43					
Recto-bulbar fistula, n = 8	NA	8 (18.6)	6.5 (5.0–7.0)	4 (26.7)	2 (9.5)
Recto-prostatic fistula, n = 14	NA	14 (32.6)	6.5 (1.8–16.3)	5 (33.3)	9 (42.9)
Type not specified, n = 21	NA	21 (48.8)	10.0 (6.0–13.5)	6 (40.0)	10 (47.6)
Imperforate anus without fistula, n = 15	2 (1.6)	13 (9.8)	8.0 (4.0–16.0)	9 (11.0)	4 (26.7)
Complex					
Recto-vesical fistula, n = 6					
Recto-vesical fistula, n = 3	NA	3 (50.0)	12.0 (2.0-NA)	1 (50.0)	1 (33.3)
Recto-bladderneck fistula, n = 3	NA	3 (50.0)	2.0 (0.0-NA)	1 (50.0)	2 (66.7)
Cloaca, n = 14					
Short common channel, n = 11	11 (91.7)	NA	13.0 (5.0–16.0)	5 (62.5)	7 (70.0)
Long common channel, n = 1	1 (8.3)	NA	19.0 (19.0–19.0)	1 (12.5)	1 (10.0)
Cloacal extrophia, n = 1	0	1 (50.0)	0.0 (0.0–0.0)	1 (12.5)	1 (10.0)
Type not specified, n = 1	0	1 (50.0)	0.0 (0.0–0.0)	1 (12.5)	1 (10.0)
Rare/regional variants, n = 9					
Pouch colon, n = 1	0	1 (25.0)	9.0 (9.0–9.0)	1 (25.0)	1 (20.0)
Colonic atresia, n = 1	0	1 (25.0)	13.7 (13.7–13.7)	0 (0.0)	1 (20.0)
Rectal atresia, n = 3	1 (20.0)	2 (50.0)	4.0 (1.0-NA)	1 (25.0)	1 (20.0)
Recto-vaginal fistula, n = 3	3 (60.0)	NA	16.0 (4.0-NA)	1 (25.0)	2 (40.0)
H-fistula, n = 1	1 (20.0)	NA	1.0 (1.0–1.0)	1 (25.0)	0
Unknown, n = 3	0	3 (2.3)	14.0 (0.0-NA)	2 (2.4)	1 (33.3)
**Total, n = 254**	122 (48.0)	132 (52.0)	7.0 (3.0–12.0)	82 (32.3)	66 (26.0)

Bold indicates the total number of patients

*ARM* anorectal malformation. *n* number. *IQR* interquartile range. *NA* not applicable. *VACTERL* VACTERL-association (i.e. vertebral, anorectal, cardiac, tracheo-esophageal, renal and limb anomalies)

*Syndromic anomalies included various syndromes such as Cat-eye syndrome, Cri-du-chat syndrome, Caudal regression syndrome, Trisomy 21, Currarino, and more

### Imaging studies

In total, 234 patients (92.1%) underwent additional imaging studies (i.e. US and/or MRI) to identify SCA at any moment in time (i.e. prior to or after ARM correction surgery). Over the 3 time periods, percentage of screening with US was lower in the second period, whereas period 1 and 3 are similar (91.8% vs. 77.4% vs. 91.8%, p-trend = 0.017). Over the 3 time periods, percentage of screening with MRI decreased (37.6% vs. 22.6% vs. 17.6%, p-trend = 0.009). Some 168 patients (66.1%) underwent solely US, 13 patients (5.1%) underwent solely MRI, and in 53 patients (20.9%), both US and MRI were performed. In 20 patients (7.9%), data on additional imaging studies was missing.

### Spinal cord anomalies

In total, SCA were identified in 52 of 234 patients (22.2%) that underwent screening with additional imaging studies (i.e. US and/or MRI). Over the 3 time periods, percentage of identified SCA remained similar (18.8% vs. 22.6% vs. 20.0%, p-trend = 0.654). In the SCA patients group, 13 patients (25.0%) underwent solely US, 3 patients (5.8%) underwent solely MRI, and in 36 patients (69.2%), both US and MRI were performed. SCA were identified through US in 42 patients (80.8%), with median age at diagnosis of 2.0 days (IQR 1.0–7.3). With US, FT anomalies were diagnosed most often in 21 patients (50.0%). SCA were identified through MRI in 32 patients (61.5%) with median age at diagnosis of 8.5 weeks (IQR 1.0–39.0). With MRI, FT anomalies and conus anomalies were diagnosed most often, in 16 patients (50.0%) and 11 patients (34.3%), respectively. In 8 patients (15.4%), SCA were identified with MRI where US findings were undetermined or in of unclear anatomy (n = 7) or data was missing (n = 1) (i.e. FT anomaly (n = 3), conus anomaly (n = 3), hydromyelia (n = 3), TSC (n = 1), caudal regression syndrome (n = 1), and spinal lipoma (n = 1)). Overall, SCA were most often identified in patients with cloacal malformations (6 of 14 patients (42.9%)) and rare/regional variants (4 of 9 patients (44.4%)). An overview of number of patients with SCA identified though imaging studies according to type of ARM is shown in Tables 2 and 3. In multivariable analysis, presence of syndromes or VACTERL-association (OR 2.56, 95% CI 1.31–4.99, p = 0.006) and complex ARM type (OR 3.61, 95% CI 1.24–10.48, p = 0.018) were independently associated with SCA (see Table 4).

**Table 2:** Number of patients with SCA identified through sc-US according to type of ARM

Type of ARM	sc-US		SCA	
	n^a^ (%)	n^b^ (%)	TSC	Other*
			n (%)	n (%)
Recto-perineal fistula, n = 109	95 (87.2)	7 (7.4)	2 (28.6)	5 (71.4)
Recto-vestibular fistula, n = 49	46 (93.9)	10 (21.7)	2 (20.0)	8 (80.0)
Recto-urethral fistula, n = 43				
Recto-bulbar fistula, n = 8	8 (100.0)	1 (12.5)	1 (100.0)	–
Recto-prostatic fistula, n = 14	14 (100.0)	4 (28.6)	2 (50.0)	2 (50.0)
Type not specified, n = 21	15 (71.4)	4 (26.7)	2 (50.0)	2 (50.0)
Recto-vesical fistula, n = 6				
Recto-vesical fistula, n = 3	3 (100.0)	1 (33.3)	–	1 (100.0)
Recto-bladderneck fistula, n = 3	3 (100.0)	1 (33.3)	–	1 (100.0)
Cloaca, n = 14				
Short common channel, n = 11	9 (81.8)	3 (33.3)	1 (33.3)	2 (66.7)
Long common channel, n = 1	1 (100.0)	1 (100.0)	–	1 (100.0)
Cloacal extrophia, n = 1	1 (100.0)	1 (100.0)	–	1 (100.0)
Type not specified, n = 1	1 (100.0)	1 (100.0)	–	1 (100.0)
Anal stenosis, n = 6	5 (83.3)	2 (40.0)	–	2 (100.0)
Imperforate anus without fistula, n = 15	14 (93.3)	3 (21.4)	–	3 (100.0)
Rare/regional variants, n = 9				
Pouch colon, n = 1	–	–	–	–
Colonic atresia, n = 1	–	–	–	–
Rectal atresia, n = 3	3 (100.0)	1 (33.3)	1 (100.0)	–
Recto-vaginal fistula, n = 3	2 (66.7)	2 (100.0)	1 (50.0)	1 (50.0)
H-fistula, n = 1	–	–	–	–
Unknown, n = 3	1 (33.3)	–	–	–
**Total, n = 254**	221 (87.0)	42 (19.0)	12 (28.6)	30 (71.4)

Bold indicates the total number of patients

*ARM* anorectal malformation. *SCA* spinal cord anomalies. *n* number. *sc-US* spinal cord ultrasound. *n*^*a*^ number of patients that underwent sc-US. *n*^*b*^ number of patients that had SCA identified with sc-US. *TSC* tethered spinal cord

*Other included various spinal cord anomalies (e.g., filum terminale anomalies, spinal lipoma, ventriculus terminalis)

**Table 3:** Number of patients with SCA identified through sc-MRI according to type of ARM

Type of ARM	sc-MRI		SCA	
	n^c^ (%)	n^d^ (%)	TSC	Other*
			n (%)	n (%)
Recto-perineal fistula, n = 109	15 (13.8)	6 (40.0)	1 (16.7)	5 (83.3)
Recto-vestibular fistula, n = 49	14 (28.6)	7 (50.0)	4 (57.1)	3 (42.9)
Recto-urethral fistula, n = 43				
Recto-bulbar fistula, n = 8	2 (25.0)	2 (100.0)	–	2 (100.0)
Recto-prostatic fistula, n = 14	5 (35.7)	3 (60.0)	1 (33.3)	3 (66.7)
Type not specified, n = 21	8 (38.0)	4 (50.0)	–	4 (100.0)
Recto-vesical fistula, n = 6				
Recto-vesical fistula, n = 3	1 (33.3)	–	–	–
Recto-bladderneck fistula, n = 3	1 (33.3)	1 (100.0)	1 (100.0)	–
Cloaca, n = 14				
Short common channel, n = 11	5 (45.5)	1 (20.0)	1 (100.0)	–
Long common channel, n = 1	1 (100.0)	1 (100.0)	–	1 (100.0)
Cloacal extrophia, n = 1	–	–	–	–
Type not specified, n = 1	1 (100.0)	1 (100.0)	–	1 (100.0)
Anal stenosis, n = 6	3 (50.0)	1 (33.3)	1 (100.0)	-
Imperforate anus without fistula, n = 15	4 (26.7)	2 (50.0)	–	2 (100.0)
Rare/regional variants, n = 9				
Pouch colon, n = 1	1 (100.0)	1 (100.0)	1 (100.0)	-
Colonic atresia, n = 1	1 (100.0)	–	–	–
Rectal atresia, n = 3	1 (33.3)	–	–	–
Recto-vaginal fistula, n = 3	1 (33.3)	1 (100.0)	1 (100.0)	–
H-fistula, n = 1	–	–	–	–
Unknown, n = 3	2 (66.7)	1 (50.0)	–	1 (100.0)
**Total, n = 254**	66 (26.0)	32 (48.5)	11 (34.4)	21 (65.6)

Bold indicates the total number of patients

*ARM* anorectal malformation. *SCA* spinal cord anomalies. *n* number. *sc-MRI* spinal cord magnetic resonance imaging. *n*^*c*^ number of patients that underwent sc-MRI. *n*^*d*^ number of patients that had SCA identified with sc-MRI. *TSC* tethered spinal cord

*Other included various spinal cord anomalies (e.g., filum terminale anomalies, spinal lipoma, ventriculus terminalis)

**Table 4:** Uni- and multivariable logistic regression for the association between baseline characteristics and spinal cord anomalies

	Univariable		Multivariable	
	OR (95% CI)	p-value	OR (95% CI)	p-value
Sexe				
Female	Ref		Ref	
Male	1.09 (0.59–2.01)	0.788	1.05 (0.54–2.04)	0.878
Underlying VACTERL-association or genetic syndrome*				
Not present	Ref		Ref	
Present	2.88 (1.59–5.58)	< 0.001	2.56 (1.31–4.99)	0.006
Vertebral anomaly				
Not present	Ref		Ref	
Present	3.20 (1.69–6.09)	< 0.001	1.73 (0.82–3.65)	0.149
ARM type				
Simple ARM types	Ref		Ref	
Intermediate ARM types	2.47 (1.19–5.11)	0.015	1.91 (0.89–4.12)	0.099
Complex ARM types	5.77 (2.24–14.85)	< 0.001	3.61 (1.24–10.48)	0.018

*OR* Odds Ratio. *CI* confidence interval. *VACTERL* VACTERL-association (i.e. vertebral, anorectal, cardiac, tracheo-esophageal, renal and limb anomalies). *ARM* anorectal malformation

*Genetic syndrome included various syndromes such as Cat-eye syndrome, Cri-du-chat syndrome, Caudal regression syndrome, Trisomy 21, Currarino, and more

### Tethered spinal cord

In total, TSC was diagnosed by imaging studies (radiological TSC) in 19 of 234 screened patients (8.1%) with median age at diagnosis of 3 days (IQR 2.0–8.0). Within the SCA patient group, over the 3 time periods percentage of identified radiological TSC increased (31.3% vs. 36.8% vs. 41.2%, p-trend = 0.558). Radiological TSC was identified in 12 patients (63.2%), without the presence of symptoms during complete follow-up (i.e. without any symptoms potentially associated to TSC). In 7 of 19 patients (36.8%) symptoms associated to TSC were identified (i.e. sensibility and motor loss (n = 1) and increased reflexes (n = 1), in addition to neurogenic bladder disorder (n = 7)). The sensibility and motor loss were present since birth without progression over time. Therefore, no surgical intervention was performed. In 5 patients (26.3%), only a pediatric neurologist was consulted for determining treatment strategy, and in 11 patients (57.9%) both a pediatric neurologist and neurosurgeon were consulted. Consequently, 4 of 19 patients (21.1%) underwent neurosurgical intervention for TSC. The indication for neurosurgical intervention were bladder dysfunction and increased reflexes in 1 patient, bladder dysfunction in 1 patient, and the nature of the anomaly as seen on additional imaging studies in 2 patients. Symptoms associated to TSC were present in only 2 patients (50.0%) that underwent surgery and resolved postoperatively. Neurosurgical intervention included terminal filum cleaving (i.e. untethering) in all 4 patients at ages 1 year (n = 2), 2 years, and 7 years respectively. No complications after untethering surgery were encountered. In total, 15 asymptomatic patients (78.9%) underwent conservative (wait-and-see) treatment, without onset or progression of symptoms, and follow-up ranging from 3 to 16 years. An overview of the identified patients with TSC can be found in Table 5.

**Table 5:** Characteristics of patients with ARM and TSC

Type of ARM	Sexe	Age at diagnosis	Diagnostic medium	Symptoms	Treatment strategy	Follow-up*
Recto-perineal fistula	Male	2 days	US	No	Conservative	No
Recto-perineal fistula	Male	5 days	US, MRI	Yes, bladder dysfunction	Conservative	Yes, once
Recto-urethral fistula	Male	0 days	US, MRI	Yes, bladder dysfunction	Conservative	Yes, interval unknown
Recto-urethral fistula	Male	1 day	US	Yes, bladder dysfunction and increased reflexes	Operative	Yes, every year
Recto-urethral fistula	Male	2 days	US	No	Operative	Yes, interval unknown
Recto-urethral fistula	Male	8 days	US	No	Operative	Yes, interval unknown
Recto-urethral fistula	Male	9 days	US	No	Conservative	Yes, once
Recto-vesical fistula	Male	9 days	MRI	No	Conservative	Yes, interval unknown
Rare/regional variants	Male	6 days	US	No	Conservative	No
Rare/regional variants	Male	25 days	MRI	Yes, bladder dysfunction and stable sensibility and motor function loss	Conservative	Yes, every year
Anal stenosis	Female	4 years	MRI	No	Conservative	Yes, interval unknown
Recto-vestibular fistula	Female	1 day	US	No	Conservative	Yes, every year
Recto-vestibular fistula	Female	2 days	MRI	No	Conservative	Yes; interval unknown
Recto-vestibular fistula	Female	2 days	US	No	Conservative	No
Recto-vestibular fistula	Female	3 days	US, MRI	No	Conservative	Yes, every year
Recto-vestibular fistula	Female	5 days	MRI	Yes, bladder dysfunction	Operative	Yes, interval unknown
Recto-vestibular fistula	Female	6 days	MRI	Yes, bladder dysfunction	Conservative	Unknown
Cloaca	Female	2 days	US, MRI	Yes, bladder dysfunction	Conservative	Yes, every year
Rare/regional variants	Female	1 day	US, MRI	No	Conservative	Yes, every year

*ARM* anorectal malformation. *TSC* tethered spinal cord. *US* ultrasound. *MRI* magnetic resonance imaging

*Follow-up by a pediatric neurologist and/or neurosurgeon

## Discussion

In our cohort of 254 ARM patients, approximately one fifth of the patients had a spinal cord anomaly; both patients with simple ARM types, as well as patients with more complex ARM types. In the majority of patients, our screening protocol with US and/or MRI was followed. SCA were identified solely with US in 39%, solely with MRI in 19%, and with US and MRI in 42% of the patients. Furthermore, in 15% of patients, SCA were identified on MRI where US findings were undetermined or of unclear anatomy. Radiological TSC was present in 19 patients, of whom 12 were asymptomatic and 7 symptomatic. In total, 4 patients with radiological TSC underwent uncomplicated neurosurgical intervention which resolved symptoms in 2 symptomatic patients.

This study showed that SCA were present in 22% of ARM patients. In the literature, the prevalence of SCA in ARM patients ranged from 26 up to 60%, which is higher than in our study [13, 14, 18]. In contrast to our study, some other studies included vertebral anomalies as SCA or solely used MRI as screening method, which potentially led to a higher prevalence in these studies compared to ours [17, 18]. Still, prevalence in studies without a standard screening protocol may be low due to underdiagnoses and missing data, and the lack of documentation on whether additional imaging studies were performed. In our study, ventriculus terminalis was included as SCA while there is a discussion on whether this finding falls within the normal variants due to lack of associated symptoms [26]. In that case, the prevalence in our study would even be lower (i.e. 20.5%) if the patients with solely ventriculus terminalis (n = 4) were excluded.

In multivariable analysis, presence of syndromes or VACTERL-association and complex ARM type were shown to be independently associated with SCA. In line with our finding, a previously performed study showed higher prevalence of SCA in ARM patients with VACTERL-association, as well as in ARM patients with vertebral anomalies [14]. Furthermore, another study found a significant correlation between the level of ARM and the presence of SCA. However, in contrast to our study, level of ARM was according to the Wingspread classification instead of the Krickenbeck, which could lead to identifying different associations [18]. Despite the associations found in both our and earlier studies, SCA were identified in patients with any ARM type, and therefore screening for SCA should be performed in all ARM children.

Radiological TSC was diagnosed in 8% of the ARM patients that underwent additional imaging studies, which is low compared to previous studies, as numbers on prevalence of TSC in ARM patients ranged widely from 6 to 65% [13, 27, 28]. Over time, TSC identification has slightly increased. This might be explained due to the improvement of US technique over time. In contrast to other studies, we used a strict definition of TSC, which can explain a higher prevalence in other studies [28]. However, due to our strict definition, TSC prevalence might be underestimated in our study, emphasizing the need for uniform international definitions. Moreover, due to the lack of a uniform international definition of TSC, it is difficult to evaluate if our TSC definition is too strict and might result in false negative outcome in the screening and thus missed cases. In addition, in our opinion, also patients with solely a low-lying conus (i.e. conus lysing below L2, one of the TSC criteria) might benefit from a consult and/or follow-up by a pediatric neurologist and urologist, and on indication repeated imaging studies, to avoid false negative outcome. Furthermore, symptomatic TSC can occur due to excessive tension on the spinal cord, which is the main reason to perform surgery (i.e. untethering) [11]. Yet, in our cohort, only 2 of 7 symptomatic patients underwent surgery. This might be explained by the thought that bladder dysfunction might not improve, and potentially even decrease due to scar tissue, after untethering surgery [29]. However, various opinions exist upon whether bladder function might improve after untethering surgery as some studies state it may be beneficial, while others state it is not [21, 29]. Furthermore, in our cohort, bladder dysfunction might be not completely due to TSC alone, as it was present in some complex and syndromic children. This is likely a possible reason for a low percentage of performed untethering surgery in symptomatic patients in our cohort (2/7, 28.6%). Regarding the patient with sensibility and motor function loss, these symptoms were present since birth and did not increase over time. Therefore, no surgical intervention was deemed necessary.

In this 21 year cohort, a vast majority (92%) of ARM patients underwent additional imaging studies to identify SCA at any moment in time. Over time, screening with US was lower in period 2 (77.4%) compared to period 1 and 3 (both 91.8%). This might be due to a lack of registration and/or missing of US images in medical records. Furthermore, screening with MRI decreased significantly over the 3 time periods, which might be explained by the fact that US was performed more often, and pediatric radiologists became more experienced in US. Median age of SCA diagnosis differed between US (i.e. 2.0 days) and MRI (i.e. 8.5 weeks), which might be explained by the fact that in AmsterdamUMC since late 2014, US was implemented as standard initial screening for SCA in ARM patients ≤ 3 months of age. Compared to MRI, US is likely more accurate for SCA diagnosis in these young patients as US provides better resolution and dynamic imaging, as the mobility of the conus could be better assessed [19]. Furthermore, in children between 6 months and 6 years of age, MRI sometimes requires anesthesia to prevent artifacts due to motion. However, advantages of MRI might be accurate visualization of the complete spinal cord, and identification of SCA in more detail. Consequently, based on our data no decisive statement can be made on whether US or MRI should be the golden standard for SCA identification [18, 19]. In our study, 8 patients had SCA identified with MRI where US findings were undetermined or of unclear anatomy (n = 7) or data was missing (n = 1) which does not support the thought that US should be the golden standard for SCA identification in patients with ARM [13]. In these patients, additional MRI was performed due to uncertainties on the ultrasound (n = 6), and the presence of an underlying syndrome (n = 2). Furthermore, SCA did have treatment consequences in some patients (e.g., excision of a meningocele, and more intensive follow-up by a pediatric neurologist in TSC and caudal regression syndrome patients). Therefore, our study group aims to perform future studies assessing the effect of the screening protocol for SCA on a change in management and the outcomes for these patients.

This study should be interpreted in the light of some strengths and limitations. First, this a large cohort from the past 21 years of patients with an extremely rare disease (i.e. ARM and TSC). Second, this study shows the data of a successfully implemented screening with US and/or MRI. Third, the consultation of a multidisciplinary team in case of uncertainties on classification of SCA improved the quality of the categorization and assessment of the encountered anomalies in this study. However, this study is prone to several limitations, of which most importantly information and registration bias, especially due to the large timeframe in which the study was executed. To reduce this to a minimum, data were extracted by two independent researchers (CdB, JG), with a data check by other coauthors (RG, CK, MS). Furthermore, due to the implementation of electronic patient records since late 2016, screening for SCA might be performed, or symptoms according to SCA and/or TSC might be present, but not adequately documented. In addition, if documentation on screening for SCA was missing, for analysis it was classified as ‘not performed’. Therefore, the incidence of SCA in our ARM population might be higher.

In conclusion, 22% of ARM patients had a spinal cord anomaly. TSC was present in 8% of patients (n = 19), of whom 4 patients underwent uncomplicated neurosurgical intervention. Screening for SCA was performed in the majority of ARM patients and improved over time. More complex types of ARM and the presence of a syndromic anomaly or VACTERL-association were shown to be associated to the presence of SCA, but SCA were identified in both patients with simple ARM types, as well as patients with more complex ARM types. Therefore, this study emphasizes the importance of screening for SCA in patients with ARM, regardless of ARM type.

## Data Availability

All data generated or analysed during this study are included in this published article (and its supplementary information files).

## Abbreviations

ARM: Anorectal malformation
ARM: Anorectal malformation
SCA: Spinal cord anomalies
TSC: Tethered spinal cord
FT: Filum terminale
US: Spinal cord ultrasound
MRI: Spinal cord magnetic resonance imaging
